# Supplementary material for: Comparison of Methods for Incorporating Related Data When Developing Clinical Prediction Models: A Simulation Study
Source: Biom J. 2026 Aug 27;68(5):e70166. doi: 10.1002/bimj.70166 (PMC13520826; doi:10.1002/bimj.70166)
Supplement: Supplementary file 2 [file BIMJ-68-e70166-s001.zip › third submission code review - R2/reproducibility_supplement/results/Plots/Individual_plots/numpredictors_plot_no_shift_SC_ID1_TS0.25_DS4_model4.pdf]

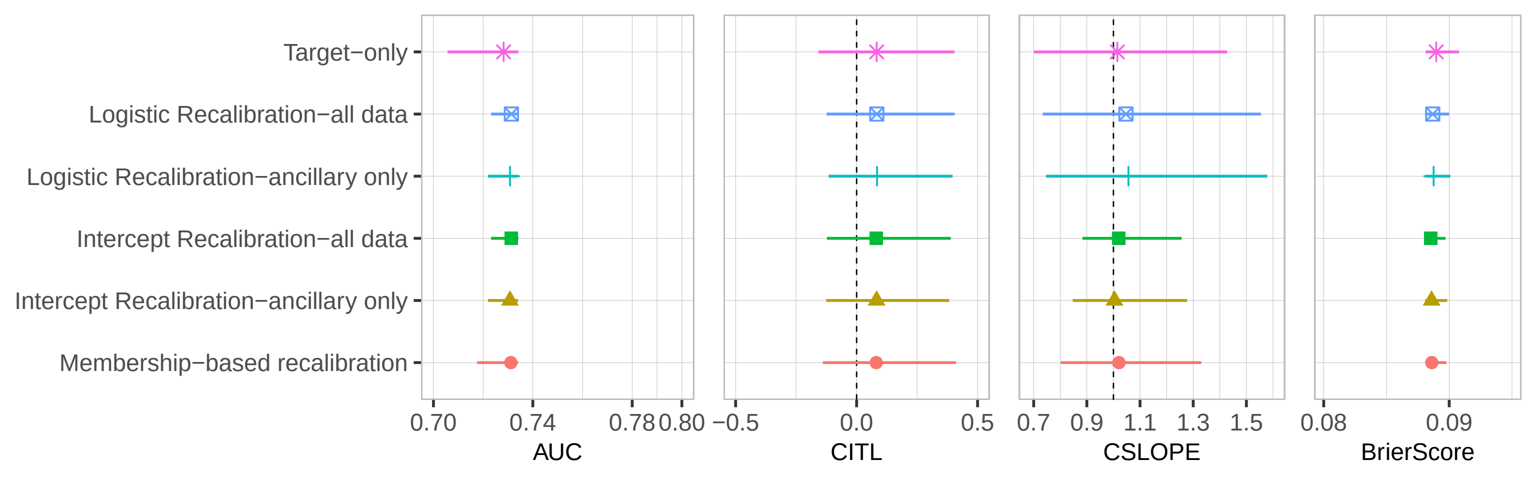

Target−only
Logistic Recalibration−all data
Logistic Recalibration−ancillary only
Intercept Recalibration−all data
Intercept Recalibration−ancillary only
Membership−based recalibration
0.70
0.74
0.78
0.80
AUC
−0.5
0.0
0.5
CITL
0.7
0.9
1.1
1.3
1.5
CSLOPE
0.08
0.09
BrierScore
